# Influence of Natural Thermal Gradients on Whole Animal Rates of Protein Synthesis in Marine Gammarid Amphipods

**DOI:** 10.1371/journal.pone.0060050

**Published:** 2013-03-27

**Authors:** Samuel P. S. Rastrick, Nia M. Whiteley

**Affiliations:** School of Biological Sciences, College of Natural Sciences, Bangor University, Bangor, Gwynedd, United Kingdom; University of Guelph, Canada

## Abstract

Although temperature is known to have an important effect on protein synthesis rates and growth in aquatic ectotherms held in the laboratory, little is known about the effects of thermal gradients on natural populations in the field. To address this issue we determined whole-animal fractional rates of protein synthesis (*k_s_*) in four dominant species of gammarid amphipods with different distributions along the coasts of Western Europe from arctic to temperate latitudes. Up to three populations of each species were collected in the summer and *k_s_* measured within 48 h. Summer *k_s_* values were relatively high in the temperate species, *Gammarus locusta*, from Portugal (48°N) and Wales (53°N) and were maintained across latitudes by the conservation of translational efficiency. In sharp contrast, summer *k_s_* remained remarkably low in the boreal/temperate species *G. duebeni* from Wales, Scotland (58°N) and Tromsø (70°N), probably as a temporary energy saving strategy to ensure survival in rapidly fluctuating environments of the high intertidal. Values for *k_s_* increased in acclimated *G. duebeni* from Scotland and Tromsø showing a lack of compensation with latitude. In the subarctic/boreal species, *G. oceanicus,* summer *k_s_* remained unchanged in Scotland and Tromsø but fell significantly in Svalbard (79°N) at 5°C, despite a slight increase in RNA content. At 79°N, mean *k_s_* was 4.5 times higher in the circumpolar species *G. setosus* than in *G. oceanicus* due to a doubling in RNA content. The relationship between whole-animal protein synthesis rates and natural thermal gradients is complex, varies between species and appears to be associated with local temperatures and their variability, as well as changes in other environmental factors.

## Introduction

Protein turnover is of prime importance to all living organisms as it represents the continuous synthesis and breakdown of proteins [1], [2]. Protein synthesis functions to repair and replace existing proteins and to synthesise or resynthesize new proteins for growth and reproduction. Protein breakdown or degradation, on the other hand, serves to remove proteins that are denatured or potentially harmful. The balance between protein synthesis and degradation determines protein retention and therefore growth, and maintains the protein pool in a constant state of flux [1]–[4]. As rates of protein synthesis and degradation are energetically costly and represent a major contribution to basal metabolic rate, protein turnover correlates closely with maintenance costs, growth efficiencies and growth rates [2], [4]. In aquatic ectotherms, for instance, faster growth rates are associated with a reduction in protein turnover rates via a reduction in degradation rates, leading to greater protein retention and lower energy expenditure [1]–[5]. Conversely, slower growth rates are associated with elevated rates of protein turnover leading to less protein retention, but increased energy expenditure. Elevated rates of protein synthesis are also associated with the increased ability of ectotherms to survive environmental perturbations by allowing for protein remodelling, flexibility in enzyme production and the repair of damaged proteins [1], [2], [6].

Most of our understanding of the effects of environmental change on protein turnover in aquatic ectotherms comes from studies on the effects of temperature on protein synthesis rates. This relationship, however, is complicated and depends on previous thermal histories (natural thermal habitat), rates of temperature change, and thermal experiences (acclimation vs acclimatisation with acclimation referring to exposure to a change in temperature under otherwise constant conditions in the laboratory, and acclimatisation referring to exposure to multiple changes in the natural environment) [4], [7]. Studies on continually fed individuals from a single species of similar body size demonstrate that whole-animal rates of protein synthesis increase with acclimation temperature [8]–[11]. Species from polar habitats, however, have considerably lower protein synthesis rates than temperate species when standardised for body mass and measured at their respective habitat temperatures [4]. The reduction in protein synthesis rates in polar species occurs despite compensatory increases in both RNA capacity, and in some cases, translational efficiencies [12], [13], and is thought to be influenced by the direct effects of temperature and seasonal restrictions in food supply. It has also been suggested that ectotherms show a reduced ability to synthesise proteins in the cold (<5°C), as low temperatures are detrimental for the formation and maintenance of native protein structures leading to the continuous degradation of proteins [4]. Evidence for the presence of a thermal constraint on protein synthesis at very low temperatures comes from measurements of protein synthesis rates and growth retention efficiencies in the Antarctic limpet, as well as studies on the production of heat shock proteins in Antarctic fish. Studies in the Antarctic limpet (*Nacella concinna*) demonstrate that protein retention efficiencies are lower than those measured in temperate and tropical species, suggesting that a greater proportion of the protein synthesised is degraded in the species living in the extreme cold [14], [15]. In field acclimatised Antarctic fish (*Trematomus bernachii*), heat shock protein 70 was expressed at high levels and ubiquitin-conjugated protein levels were elevated, further supporting the suggestion that extremely low temperatures have a denaturing effect [16]. Decreases in protein synthesis rates and protein stability in the extreme cold could account for the slow growth rates reported in many adult polar marine species [4], [17], [18], and the low protein retention efficiencies observed by [15].

Studies on stenothermal and eurythermal ectotherms living in the northern hemisphere can be used to further our understanding of the relationship between whole-animal protein synthesis rates and thermal habitat. Examination of northern over southern polar ectotherms has several advantages in that comparisons can be made within and between closely-related species from arctic, sub-arctic and boreal regimes to standardise for phylogenetic diversity and life-style. Such an approach also allows for an examination of cold-water species living in more variable temperature regimes [19], [20]. The current study focused on gammarid amphipod crustaceans, which are widely distributed along the coastal fringes of Western Europe where they are found in abundance throughout the sublittoral and intertidal zone from Southern Spain to Svalbard, north of the Arctic Circle. Gammarids play a key role in the structure and function of aquatic communities, and as primary consumers, have a significant impact on the transfer of carbon up the food chain [21], [22]. Species differ in latitudinal distribution patterns and position on the shore [20]. They experience different thermal habitats, show a range of physiological tolerances to environmental change, and demonstrate marked differences in growth rates and life history traits with latitude [23]–[26].

**Figure 1:**
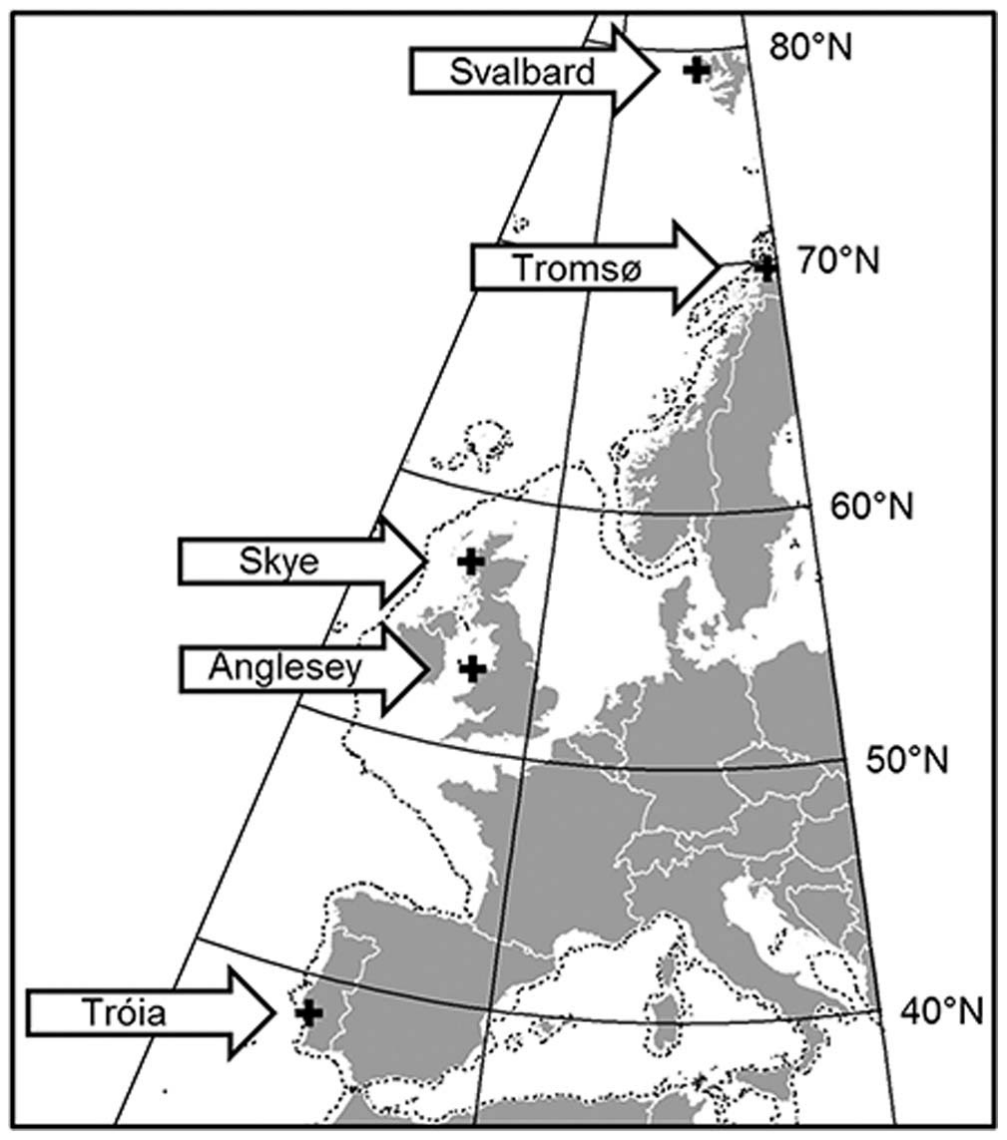
Map of Western Europe to show the location of the various collection sites used in the study. Further details are given in Table 1.

The current study focused on four gammarid species which are dominant in the northeastern Atlantic and arctic regions: the arctic/subarctic species, *Gammarus setosus*; the subarctic/boreal species, *G. oceanicus*; the boreal/temperate species, *G. duebeni* and the temperate species, *G. locusta* (Fig. 1; Table 1). All species, apart from *G. duebeni* inhabit the low intertidal, although when they coexist, *G. setosus* is slightly higher on the shore than *G. oceanicus* [27]. *G. setosus* is a circumpolar, cold-water species which prefers temperatures of <4°C [27], [28]. The remaining 3 species are more eurythermal, although the low intertidal species *G. oceanicus* and *G. locusta*, are less tolerant to temperature change than the high intertidal species, *G. duebeni*, which is well adapted to highly variable temperatures [23]. A broader tolerance to temperature change, in this case acute temperature change, in amphipods living in more variable environments has also been observed by [29], [30]. All four gammarid species are tolerant of salinity change (euryhaline) but to differing degrees with *G. duebeni* showing the greatest tolerance to seawater dilution [25]. Differences in life-history traits have also been observed between the study species. For instance, the high intertidal species, *G. duebeni*, with the highest tolerances to temperature and salinity have smaller body sizes at maturity than the less tolerant, low intertidal species *G. oceanicus* when measured at the same latitude (R. Crichton, unpublished observations). Gammarid amphipods also show intraspecific differences in life-history traits across a latitudinal cline with high latitude amphipods characterised by larger body sizes, delayed maturity, single broods with larger eggs, and biannual or perennial life stages [24].

**Table 1:** Location of collection sites and species sampled.

Collection site	Latitude and longitude	Species	Temperature (°C)*	Day length*
Ny-Ålesund, Svalbard	78.92°N, 11.92°E	*G. oceanicus* *G. setosus*	55	24L:0D
Tromsø, Norway	69.61°N, 18.9°E	*G. oceanicus* *G. duebeni*	1015	24L:0D
Skye, Scotland	57.66°N, 5.33°W	*G. oceanicus* *G. duebeni*	1313	17L:7D
Anglesey, Wales	53.23°N, 4.51°W	*G. locusta* *G. duebeni*	1313	16L:8D
Tróia, Portugal	38.48°N, 0.88°W	*G. locusta*	21	14∶10L

* habitat temperature and day length at time of capture.

The purpose of the current study was to characterise protein synthesis rates of gammarid amphipods distributed along a latitudinal cline and occupying different thermal habitats, from cold-water, relatively stable thermal habitats to more temperate, highly variable eurythermal habitats. More specifically, we aimed to assess protein synthesis rates and associated variables (protein synthesis capacity and translational efficiencies) in summer acclimatised amphipods to further understand the responses of natural populations when temperatures and levels of nutrition are the most favourable for growth. During the course of the study, we were keen to establish whether the colder habitat temperatures at the polar latitude (79°N) limit protein synthesis rates in two species of gammarids with differing latitudinal distribution patterns: circumpolar *G. setosus* and subarctic/boreal *G. oceanicus*. We were also interested in determining whether *G. duebeni* occupying the high intertidal in highly variable thermal environments is characterised by elevated protein synthesis rates in order to synthesise, repair and replace damaged proteins. Ultimately we are interested in using this study as an initial step towards establishing whether protein synthesis rates can be used to explain the relationship between thermal environment, physiological tolerances and life-history traits in natural populations of coastal invertebrates.

## Materials and Methods

### Study System

Four gammarid species (*G. setosus*, *G. oceancius*, *G. locusta* and *G. duebeni*) and nine populations were examined in this study. *G. setosus* is circumpolar in arctic regions and is distributed as far north as land extends [28], [31]. To the south, European populations of *G. setosus* on mainland Norway have only been reported in the extreme north [32]. *G. oceanicus*, has a wider distribution range then *G. setosus* as it extends from western coasts of Svalbard in the north to Scotland in the south [25], [28]. *G. locusta* has a more southerly distribution extending from Norway to southern Portugal and Spain [33]. Further south *G. locusta* occupies the low intertidal, but further north along Norwegian coasts, *G. locusta* occupies the most exposed parts of the outer fjords and may even become sublittoral to avoid competition with *G. oceanicus* [33], [34]. *G. locusta* has not been reported in northern Norway [34], but *G. oceanicus, G. locusta* and *G. duebeni* are all found in the Baltic Sea where salinities are low but stable [35].

*G. duebeni* is a brackish water species which inhabits estuaries and areas under freshwater influence in the high intertidal where temperatures and salinity can be highly variable [25], [36]. *G. duebeni* has an exceptionally wide tolerance to salinity but prefers salinities of 5–15 psu and can be found living in freshwater for extended periods [36]. It is also more tolerant to temperature change than the low intertidal species *G. oceanicus* and *G. locusta,* which mostly prefer more marine environments [23], [25]. Little is known about ability of the cold-water species *G. setosus* to tolerate environmental change, but initial studies indicate that *G. setosus* is less tolerant than sub-arctic populations of *G. oceanicus* to elevated temperatures, but this species can occupy the mouths of melt water streams especially when under threat of increasing seawater temperatures [31], [36], [37]. Populations of *G. oceanicus* and *G. setosus* at the collection site on western Svalbard typically experience summer temperatures can range daily from 1.5 to 6°C. At the same latitude in Tromsø, daily summer habitat temperatures of 5–15°C for *G. duebeni,* and 8–11°C for *G. oceanicus.* Further south on the Isle of Skye and on Anglesey, *G. duebeni* can experience daily variations in summer habitat temperatures from 9−16.5°C. Annual habitat temperatures range from 6–16°C for *G. locusta* in Wales (53°N), and from 11–24°C for *G. locusta* in southern Portugal [33].

Life-history traits differ between the species. *G. setosus* and *G. oceanicus* at high latitudes have the largest female body sizes at maturity (20–26 cm mean body length) [26], [31], and *G. locusta* and *G. duebeni* the smallest at 8–10.0 cm [33]. Subarctic populations of *G. oceanicus* and *G. setosus* exhibit K-selected traits typical of stenothermal marine invertebrates [26]. At 79°N both species produce one brood per year along with fewer but larger eggs [26]. Further south, at 59°N, *G. oceanicus* produces 3 broods per year and the eggs are smaller but more numerous [38]. *G. oceanicus* shows both an increase in time to maturity and an increase in life-span at the higher latitudes [26], [38]. Costa and Costa [33] describe *G. locusta* in Portugal as being mulivoltine as ovigerous females are observed all year round. Similar responses have been observed in *G. locusta* and *G. duebeni* collected from Wales, whereas populations of *G. duebeni* collected from Tromsø are likely to produce limited number of broods during the spring/summer when conditions are more favourable.

### Animal Collection and Husbandry

Gammarid amphipod species were collected from five locations representing various latitudes along the coastal fringes of Western Europe from southern Portugal to Svalbard between June to September 2007 and 2008 to match those previously examined for metabolic rate [39] (Fig. 1; Table 1). The four species of interest were caught when present to give one arctic population of *G. setosus*, 3 populations of *G. oceanicus*, 2 populations of *G. locusta* and 3 populations of *G. duebeni*. The populations studied here are those that could be found on the shore at the various latitudes. *G. locusta* was not found after extensive searching at various coastal sites on the Isle of Skye. Neither *G. locusta* or *G. setosus* were found at sites close to Tromsø. The location of each collection site is given in Fig. 1, and a summary of the species and their populations is given in Table 1. Mature adult amphipods were collected from under stones and fucoids at low tide using hand nets. Moulting individuals and brooding females were excluded from experiments. Microhabitat temperatures were recorded at the time of collection using a K Type thermocouple (Hanna Instruments Inc, Michigan, USA).

### Ethics Statement

No specific permits were required for the collection of the animals at any of the sites. Field work at Ny-Ålesund was registered with Svalbard Science Forum and assessed by NERC, UK. None of the species collected in this study were endangered or protected.

### Acclimatised Amphipods

Immediately after capture, adult amphipods from all five collection sites were transported to Bangor University, UK. For transport, amphipods were wrapped in filter paper soaked in seawater and held at their appropriate capture temperatures. Transport times varied but did not exceed 24 h even in amphipods transported from Ny-Ålesund, Svalbard. On arrival at Bangor, the amphipods were held in their original seawater at their corresponding capture temperatures and light regimes to mimic natural conditions during collection (Table 1). All species, apart from *G. duebeni*, were held in full strength seawater. *G. duebeni* were held in 50% seawater which is close to their iso-osmotic point [40]. Amphipods were maintained in this way for at least 24 h to allow the animals to recover from handling and transportation effects. None of the amphipods were fed during this period. All of the protein synthesis determinations were made within 48 h of initial capture to ensure that values represented acclimatised animals.

### Acclimated Amphipods

To investigate the specific effect of temperature on protein synthesis rates, ten additional amphipods from two populations of *G. duebeni* (Tromsø, Norway and Scotland) and one population of *G. oceanicus* (Scotland) were transferred to Bangor University and acclimated to a common temperature of 10°C in either fully aerated full strength seawater (*G. oceanicus*) or 50% seawater (*G. duebeni*) for four weeks in a 12L:12D regime. A third of the seawater was replaced every week before the amphipods were fed a diet of algal fish food (Tetra*Veg*®, Tetra GmbH, Germany). Amphipods were starved for 48 h before protein synthesis rates were determined.

### Determination of Protein Synthesis Rate: Validation of Methodology

Fractional rates of protein synthesis were determined using the flooding dose method [41] modified for use in crustaceans [8], [42]. This technique relies on the administration of a single but very large dose of amino acid which floods all of the intracellular precursor pools to maintain the same level of specific radioactivity during the period of incorporation into protein. Validation of the technique was first carried out to test whether three important assumptions were met during the incorporation period in gammarid amphipods. These are: (1) the intracellular free-pools are completely flooded by the injected unlabelled phenylalanine; (2) specific radioactivities of the free-pools increase rapidly and remain stable or decline slowly over time; and (3) the increase in specific radioactivity in the protein-bound fraction is significant and linear. The first requirement of the validation process was established by injecting five *G. oceanicus* from Tromsø (70°N) and five *G. locusta* from Wales (53°N) with phenylalanine-free crab saline [43]. Phenylalanine levels in the free-pools of these individuals were then compared to those from the same population injected with the flooding dose. The remaining two criteria were tested by performing a time-course experiment in which *G. oceanicus* and *G. duebeni* from Scotland (58°N) were injected with the flooding dose and held in aerated seawater at 13°C for 0.5 h, 1 h or 2 h. An additional time course experiment was carried out on *G. oceanicus* from Ny-Ålesund (79°N) at 5°C by incubating amphipods for 1 h, 2 h or 3 h. The purpose of this additional time-course was to determine whether the lower temperatures increased the time courses for the second and third criteria.

To determine protein synthesis rates, amphipods were injected with crab saline at a dose of 2 µl 50 mg^−1^ wet body mass, containing 150 mmol l^−1^ of unlabelled L-phenylalanine and 3.7 MBq ml^−1^ of L- [2,3,4,5,6-^3^H] phenylalanine (G. E. Healthcare. Specific Activity 4.37 TBq mmol^−1^). To enable injection of small volumes (µls) of labelling cocktail, amphipods were held within a Perspex stage in 2 ml of seawater at the appropriate salinity and temperature, so that the dorsal surface protruded above the surface of the seawater and the pereopods, thoracic gills and pleopods remained submerged. Labelling cocktail was injected into the haemolymph by inserting a fine glass microcapillary between the 1^st^ and 2^nd^ pereon segments and into the bulbus arteriosus of the heart. All injections were performed under a dissecting microscope (M32, Wild Heerbrugg, Switzerland) using a micro-droplet manipulation system [44]. After delivery of the labelling cocktail, the glass microcapillary was left in place for 10 sec to ensure complete circulation of the label. Amphipods were then removed and held separately in 50 ml of fully aerated seawater at their respective capture temperatures and salinities for the appropriate incorporation period. After incubation, amphipods were sacrificed, snap frozen in liquid nitrogen and stored at −80°C.

### Determination of Protein Synthesis Rate: Analysis

Samples were analysed for protein synthesis rates according to [41] and [8]. In summary, samples were ground under liquid nitrogen and precipitated in ice-cold 2% perchloric acid (PCA). After centrifugation the resulting supernatant (free-pool fraction) was stored at −20°C and the pellet containing the protein bound-fraction was washed twice in 2% PCA and solubilised in 0.3 N NaOH for 1 hour at 37°C. The alkali-soluble protein was determined from 20 µl sub-samples using a modified Lowry method with bovine serum albumin as standard [45]. Protein and DNA were precipitated from the alkali-digest by addition of 12% PCA and the resulting acid-soluble fraction was removed for the estimation of RNA levels by ultraviolet absorption at 232 and 260 nm and verified using known standards (RNA, Sigma R 8508) [46]. The remaining protein pellet was hydrolysed in 6N HCl at 110°C for 24 h with subsequent evaporation of the acid to dryness, before being re-suspended in citrate buffer (pH = 6.3).

Phenylalanine levels were determined in both the free-pools and the protein-bound fractions by enzymatic conversion to β-phenylethylamine (PEA) using tyrosine decarboxylase (Worthington Biochemical Corporation, Lakewood, USA) [41]. Enzyme conversion efficiency was improved by sonication of each sample for 4×5 s before enzyme incubation [47], and determined for each new batch of enzyme by the inclusion of known phenylalanine standards (50, 100 and 150 nmol ml^−1^). β-Phenylethlamine was determined fluorometrically (Victor^2^ ™ Multilabel Counter, Perkin Elmer, Massachusetts, USA). The specific radioactivities of phenylalanine in the intracellular free-pool and protein-bound factions were determined by liquid scintillation (WinSpectral™ 1414 Liquid Scintillation Counter, Perkin Elmer, Massachusetts, USA) using Optiphase ‘Hisafe’ scintillant at a counting efficiency of 37%. Specific activity of phenylalanine in the free-pool and protein-bound fractions was expressed as disintegrations per min per nmole (dpm nmol^−1^) of phenylalanine.

### Calculations

Whole-animal fractional rates of protein synthesis (*k*_s_) were calculated using the equation [41]:
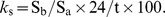
where *k_s_* =  percentage protein synthesised per day (% day^−1^); S_a_ =  specific radioactivity of phenylalanine in the intracellular free-pools (dpm nmol^−1^); S_b_ =  specific radioactivity of phenylalanine bound to protein (dpm nmol^−1^); *t* = incubation time in hours. Absolute rates of protein synthesis (*A*_s_) were calculated for each sample by multiplying the corresponding protein synthesis value by the total protein content of each whole body to give mg of protein synthesised per day. RNA to protein ratios (µg RNA mg^−1^ protein) were used to express RNA concentrations and the capacity for protein synthesis. RNA activity or translational efficiency (*K_RNA_*; mg protein mg^−1^ RNA day^−1^) was calculated using the equation from [48]:

*K_RNA_* = (10×*k*_s_/RNA:protein).

All data were standardised to 1 g wet body mass using the weight exponent 0.7 for whole animal values (*A*_s_) and −0.2 for weight-specific values (*k*_s_, *K*_RNA_, RNA:protein) [42].

### Statistical Analysis

All data were tested for normality using a Kolmgorov-Smirnov test and for homogeneity of variances using Levene’s test. As fractional rates of protein synthesis represent proportional data, all *k_s_* values were arcsine square root transformed before further analysis. For all parametric data, one-way analysis of variance (ANOVA) and the least significance difference (LSD) post-hoc test were used. Non-parameteric data were analysed using the Mann Whitney U test. Comparisons of two populations of different species inhabiting the same latitude were made using independent sample t-tests. The linear incorporation of the labelled phenylalanine into the protein-bound fraction over time was analysed using linear least-squares regression analysis. All statistical analyses were performed using SPSS software (SPSS INC., Chicago. IL, USA). All values are means ± SEM with the number of observations in parenthesis.

## Results

### Validation of Flooding Dose Methodology

**Figure 2:**
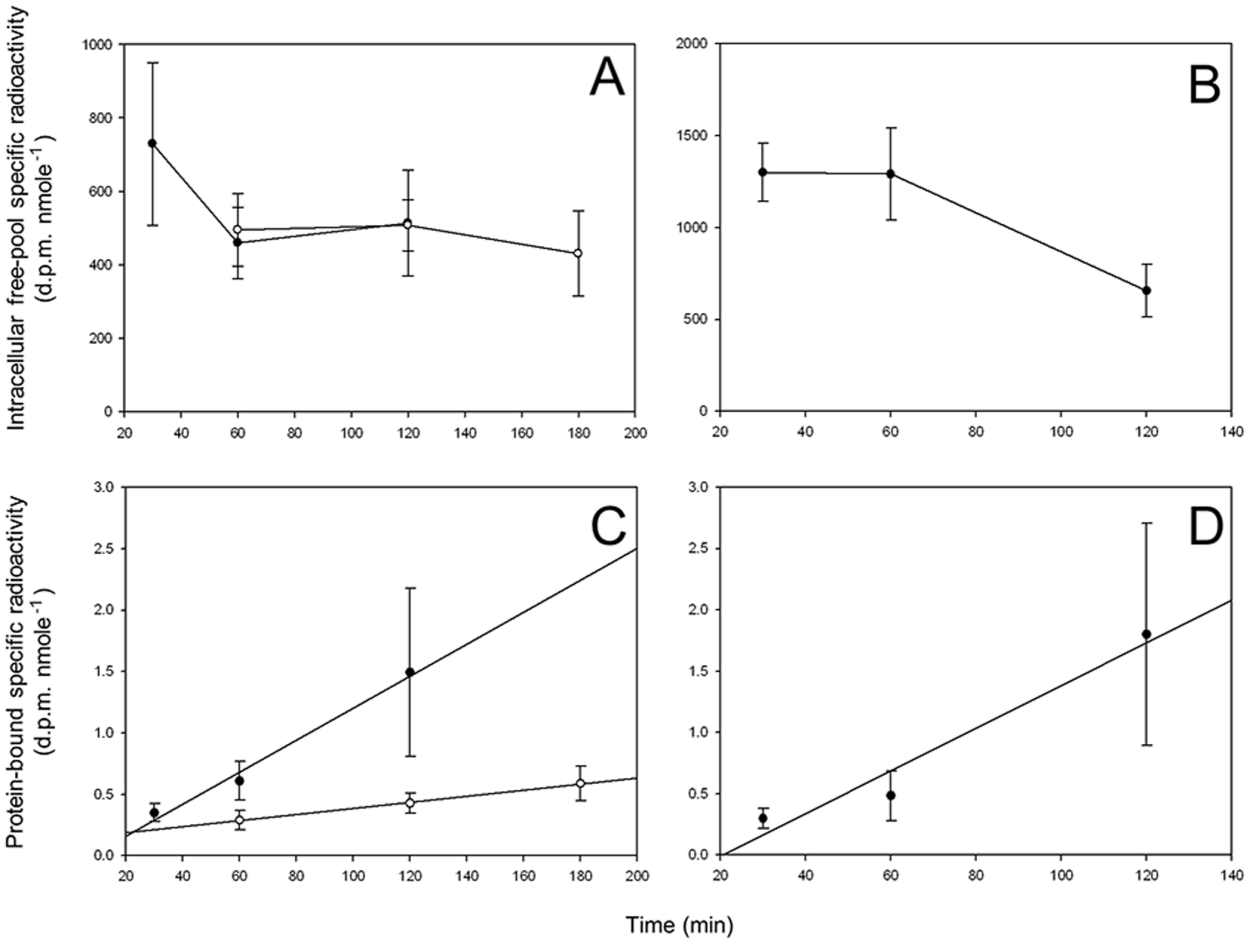
Validation of the flooding dose technique for use in gammarid amphipods. (A) Intracellular free-pool specific radioactivities for phenylalanine in *G. oceanicus* from Svalbard (79°N) held at 5°C (open circles; n = 6) and *G. oceanicus* from Scotland (58°N) held at 13°C (closed circles; n = 5). (B) Intracellular free-pool specific radioactivities for phenylalanine in *G. duebeni* from Scotland held at 13°C (n = 8). (C) The relationship between protein-bound specific radioactivities of phenylalanine and time in *G. oceanicus* from Svalbard held at 5°C (open circles; n = 6) and *G. oceanicus* from Scotland held at 13°C (closed circles; n = 5). (D) The relationship between protein-bound specific radioactivities of phenylalanine and time in *G. duebeni* from Scotland held at 13°C (n = 8). All values are means ±SEM. The equations for the linear regression lines in C and D are given in Table 2. All regression lines were highly significant and the intercepts were not significantly different to zero.

Phenylalanine levels in the intracellular free-pools increased 4.8 fold between controls (saline injection only) and the post-injection values in *G. duebeni* and *G. locusta*. This satisfies the first requirement in the validation of the flooding dose technique for use in gammarid amphipods. The second requirement of the validation process was met as the mean specific radioactivities of phenylalanine in the intracellular free-pools remained stable over the incorporation period in *G. oceanicus* from Svalbard at 5°C, as well as in *G. oceanicus* and *G. duebeni* from Scotland at 13°C. More specifically, the specific radioactivity of phenylalanine in the intracellular free-pools of *G. oceanicus* remained unchanged between 0.5 and 2 h in amphipods from Scotland at 13°C, and between 1 and 3 h in amphipods from Svalbard at 5°C (Fig. 2a). In contrast, specific radioactivities of phenylalanine in the intracellular free-pools of *G. duebeni* from Scotland at 13°C were stable between 0.5 and 1 h, but fell significantly between 1 and 2 h from 1,291±251(8) to 656±144(8) dpm nmol^−1^ phenylalanine (ANOVA, *P*<0.05; LSD, *P* = 0.028) (Fig. 2b). Consequently the stability of the free-pools in *G. duebeni* was only maintained for the first hour after injection.

Incorporation of labelled phenylalanine into protein was significant and linear between 0.5 and 2 h in both species from Scotland at 13°C, and in *G. oceanicus* from Svalbard at 5°C (Table 2; Fig. 2c,d). In each case, the intercept of the regression line was not significantly different from the origin (Table 2) indicating that the radiolabel equilibrated rapidly with the free-pools leading to the rapid incorporation of phenylalanine into proteins after injection. In *G. oceanicus*, rates of incorporation were over 5 times slower in the subarctic population from Svalbard compared with the temperate population from Scotland (Table 2). As a result the third requirement of the flooding dose technique was met in both species, and in all 3 populations. For subsequent determinations of protein synthesis rates in gammarids from all latitudes and capture temperatures, incorporation times were kept to 1 h, except for subarctic populations of *G. oceanicus* and *G. setosus* at 5°C where some animals were incubated for 2 h after injection.

**Table 2:** Least-squares regression analysis for the data presented in Fig. 2.

Species	Temp(°C)	n	b	*p*_b_	a	*p*_a_	r^2^
*G. duebeni* (58°N)	13	24	0.017±0.008	0.044	−0.36±0.65	0.585	0.17
*G. oceanicus* (58°N)	13	15	0.013±0.006	0.045	−0.10±0.46	0.832	0.26
*G. oceanicus* (79°N)	5	18	0.002±0.001	0.044	0.14±0.14	0.337	0.23

Data represents the relationship between the specific radioactivity of protein-bound phenylalanine and incorporation time as shown in Figs. 2c, d.

The regression coefficient (b) characterises the rate of incorporation of the radiolabelled amino acid into the protein bound fraction in dpm nmol phenylalanine min^−1^. *p*_b_ (*p* value) represents the significance of the least-squares regression model, and *p*_a_ represents the significance of the variation between the intercept (a) and the origin.

All vales are means ±SEM.

### Fractional and Absolute Rates of Protein Synthesis

**Figure 3:**
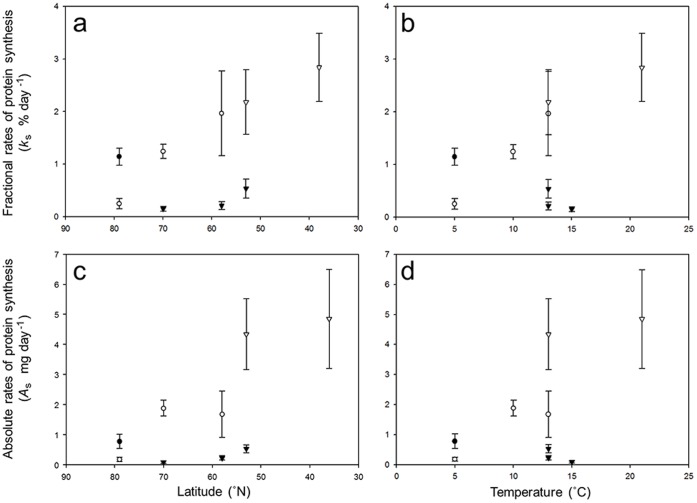
Fractional (*k*_s_; % day^−1^) and absolute (*A*_s_; mg day^−1^) rates of protein synthesis in gammarid amphipods. Values plotted as a function of latitude (a, c) or capture temperatures (b, d). Species are: *Gammarus setosus* (closed circles); *G. oceanicus* (open circles); *G. duebeni* (closed triangles) and *G. locusta* (open triangles). (a) Relationship between fractional rates of protein synthesis and latitude (y = −0.05x+3.96; r^2^ = 0.39). (b) Relationship between fractional rates of protein synthesis and capture temperature (y = 0.1x+0.02; r^2^ = 0.23). (c) Relationship between absolute rates of protein synthesis and latitude (y = −0.09x+7.32; r^2^ = 0.48). (d) Relationship between absolute rates of protein synthesis and capture temperature (y = 0.21x+−0.84; r^2^ = 0.32). Lines fitted using least-squares regression. Mean values given ±SEM. In *G. setosus*: n = 12 at 79°N. In *G. oceanicus*: n = 13 at 79°N; n = 11 at 70°N; n = 11 at 58°N. In *G. duebeni*: n = 8 at 70°N; n = 6 at 58°N; and n = 8 at 53°N. In *G. locusta*: n = 8 at 53°N; n = 7 at 38°N.

Whole-animal fractional and absolute rates of protein synthesis in the various species and their populations are plotted against latitude in Fig. 3a,c and against capture temperature in Fig. 3b,d. Latitude had a significant effect on whole-animal protein synthesis (*k_s_*) values in *G. oceanicus* as mean *k_s_* in amphipods from Svalbard was considerably lower at 0.25±0.1(11) % day^−1^ than in amphipods from Scotland at 1.96±0.8(10) % day^−1^ (ANOVA, f = 3.7, df = 2, *P*>0.05; LSD, *P*>0.05). The fall in *k_s_* with latitude was associated with an 8°C decrease in capture temperature. There was also a significant difference in absolute rates of protein synthesis (*A_s_*) in *G. oceanicus* with latitude and with temperature (ANOVA, f = 4.41, df = 2, *P*<0.05). In all cases, however, the significant fall in *k_s_* and *A_s_* occurred between populations in Tromsø and Svalbard. There were no significant changes in *k_s_* and *A_s_* between populations in Scotland and Tromsø with latitude or with temperature. In addition, there was no significant variation in whole-animal *k_s_* and *A*_s_ with latitude in either *G. locusta* or *G. duebeni.*

In Svalbard (79°N) at a common capture temperature of 5°C, *G. setosus* exhibited significantly higher whole-animal *k_s_* (t-test, t = 4.58, df = 21, *P*<0.05) and *A*_s_ values (t-test, t = 2.33, df = 20, *P*<0.05) than *G. oceanicus*. At lower latitudes, *k*_s_ and *A*_s_ values in *G. duebeni* were significantly lower than those observed in *G. oceanicus* at a common latitude of 70°N and capture temperature of 10°C (*k_s_*; t-test, t = 6.68 df = 17, *P* = <0.001) (*A*_s_; t-test, t = −5.74 df = 17, *P* = <0.001), and significantly lower than the values in *G. locusta* at 53°N and 13°C (*k*_s_; t-test, t = −2.92 df = 12, *P* = <0.05) (*A*_s_; t-test, t = −3.75 df = 12, *P* = <0.01).

After acclimation to a common temperature of 10°C for 4 weeks, there was no significant difference in mean *k*_s_ between *G. oceanicus* (1.7±0.8 (7) % day^−1^) and *G. duebeni* (0.9±0.4 (6) mg day^−1^) from Scotland (t-test, t = 1.06, df = 11 *P* = 0.31). In addition, there was no significant difference in mean acclimated *k*_s_ values between *G. duebeni* from Tromsø (1.6±0.7 (7) % day^−1^) and from Scotland (t-test, t = 0.88, df = 11, *P* = 4.21). Likewise there were no significant differences in acclimated *A_s_* between *G. oceanicus* and *G. duebeni* from Scotland and between the population of *G. duebeni* from Tromsø and the population from Scotland.

### RNA Concentrations and Activities

**Figure 4:**
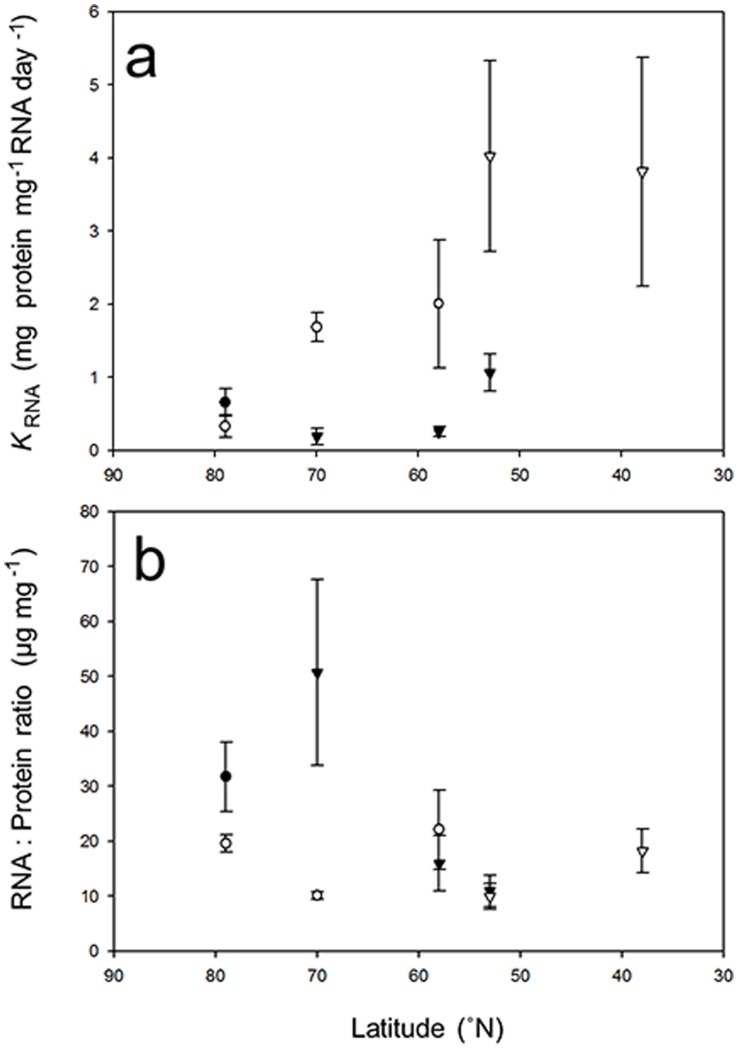
The effect of latitude on RNA activities (*K*_RNA_) and RNA concentrations (RNA: protein). Values represent: *Gammarus setosus* (closed circles); *G. oceanicus* (open circles); *G. duebeni* (closed triangles) and *G. locusta* (open triangles). (a) Relationship between *K*_RNA_ and latitude (y = −0.078x+6.38; r^2^ = 0.52). (b) Relationship between RNA:protein and latitude (y = −0.35x+0.41; r^2^ = 0.17). Lines fitted using least-squares regression. Mean values given ±SEM. In *G. setosus*: n = 12 at 79°N. In *G. oceanicus*: n = 13 at 79°N; n = 11 at 70°N; n = 11 at 58°N. In *G. duebeni*: n = 8 at 70°N; n = 6 at 58°N; and n = 8 at 53°N. In *G. locusta*: n = 8 at 53°N; n = 7 at 38°N.

RNA concentrations (RNA:protein) and activities (*K*_RNA_) are plotted against latitude in Fig. 4. *G. duebeni* and *G. oceanicus* showed significant variation in RNA: protein ratios with latitude. *G. duebeni* showed a 5-fold increase in mean values from 6.37±1.61(8) to 33.17±10.91(8) µg RNA mg^−1^ protein between populations from Wales and Tromsø (ANOVA, *f* = 6.05, *P<*0.005; LSD, *P*>0.005). *G. oceanicus* exhibited a significant increase in RNA: protein ratios between Tromsø and the most northerly population from Svalbard of 7.72±0.53(11) to 15.564±1.165(11) µg RNA mg^−1^ protein (ANOVA, *f* = 4.8, *P*<0.001; LSD, *P*>0.001). Latitude also had a significant effect on *K_RNA_* in *G. oceanicus* and *G. duebeni* but not in *G. locusta* (Fig. 4a). In *G. oceanicus*, there was a considerable drop in *K_RNA_* from 1.36±0.58(11) mg^−1^ protein mg^−1 ^RNA day^−1^ in the population from Scotland (58°N) to 0.1±0.04(11) mg^−1^ protein mg^−1 ^RNA day^−1^ in the population from Svalbard (79°N; ANOVA, *f* = 4.8, *P* = 0.05; LSD, *P*>0.05). In *G. duebeni*, *K*_RNA_ fell from 0.63±0.15(8) to 0.13±0.08(8) mg^−1^ protein mg^−1 ^RNA day^−1^ between populations from Wales (53°N) and Tromsø (70°N; ANOVA, *f* = 4.8, *P*<0.05 LSD, *P*>0.5). In *G. locusta*, *K_RNA_* remained at around 2 mg^−1^ protein mg^−1 ^RNA day^−1^ in populations from Portugal and from Wales, despite an 8°C drop in summer microhabitat temperature.

Comparisons between species collected at the same latitude revealed that *K*_RNA_ values were significantly lower in *G. duebeni* than in either *G. oceanicus* at 70°N (t-tests, t = −5.99, P<0.001), or *G. locusta* at 53°N (t-tests, t = −2.4, P<0.05). However, mean *K*_RNA_ between *G. duebeni* and *G. oceanicus* at 58°N was marginally insignificant (t-test, t = −2.06, P = 0.056). In Svalbard *G. setosus* also exhibited significantly higher *K*_RNA_ than *G. oceanicus* (t-tests, t = 3.01, P<0.05).The only significant difference in RNA:protein was observed between *G. duebeni* and *G. oceanicus* at 70°N (t-tests, t = 2.84, P<0.05) (Fig. 4b).

## Discussion

This is the first time that whole-animal fractional rates of protein synthesis have been measured between congeneric species and their natural populations distributed over a latitudinal cline from arctic, through boreal to cold- and warm-temperate regions. The species and populations of interest spanned a latitudinal range of 41°N representing a difference in summer capture temperature of 16°C between the most northerly and southerly populations. Standardisation for body size demonstrated that the highest whole-animal rates of protein synthesis in summer acclimatised amphipods were observed in the temperate species *Gammarus locusta* in Portugal (38°N) and Wales (53°N), and in the subarctic/boreal species *G. oceanicus* at the southern edge of its distribution range in Scotland (58°N). The lowest rates of summer acclimatised protein synthesis rates were observed in the arctic population of *G. oceanicus*, and in all three populations of the boreal/temperate species *G. duebeni*. Species with an intermediate rate of protein synthesis were *G. oceanicus* at boreal latitudes and the arctic population of the circumpolar amphipod, *G. setosus*. Responses also varied within species, with latitude having no effect on summer acclimatised protein synthesis rates in *G. locusta* and *G. duebeni*, but a marked effect in *G. oceanicus* at 79°N where protein synthesis rates declined. As the responses observed were species-specific, each species is considered in turn.

*G. locusta* has a more southerly distribution and is more closely related to Mediterranean gammarids than the other gammarid species investigated here [22]. At the time of collection, *G. locusta* could not be found on the Isle of Skye probably because this species is outcompeted by *G. oceanicus* and has migrated into deeper water [33], [34]. Similarly, *G. locusta* was not found on costal sites close to Tromsø because this species is unlikely to be found so far north [33]. As a result the current discussion is restricted to two populations and is therefore limited in scope. Nevertheless, the occurrence of relatively high rates of protein synthesis in summer acclimatised *G. locusta* from Portugal and Wales can be related to our previous observations on rates of growth and oxygen uptake in the same species and at the same latitudes. For instance, juvenile *G. locusta* from Wales at 53°N, have faster growth rates and reach maturity sooner than juvenile *G. duebeni* from the same latitude at a common temperature of 15°C (A. M. Posacka and S. Rastrick, unpublished data). In addition, summer acclimatised individuals from the same populations were observed to have relatively high rates of activity and energy consumption [39]. Collectively it appears that *G. locusta* from populations in Portugal and Wales are characterised by high-energy life-styles (high rates of activity), with high protein synthesis rates, faster growth rates and shorter times to maturity at relatively small body sizes. Faster growth rates suggest lower rates of protein turnover and greater protein retention [1]–[5], [49], which may help to explain why *G. locusta* is reported to be less tolerant to changes in temperature and less resistant to hypoxia than other gammarid amphipods, such as *G. oceanicus* and *G. duebeni* [23].

In contrast to *G. locusta,* summer acclimatised, whole-animal rates of protein synthesis in the high intertidal species, *G. duebeni*, were extremely low and remained low across populations in Wales, Scotland and Norway (Tromsø). Such low rates were unexpected and contradict the predication that protein synthesis rates are elevated in *G. duebeni* with its broad tolerance to changes in temperature, salinity and oxygen levels [23], [25], [40]. Alternatively the extremely low rates of protein synthesis measured in natural populations of *G. duebeni* may signify transitory responses to extreme conditions on the shore during the summer, and coincide with bouts of metabolic depression. Several intertidal invertebrates reduce their metabolic rates during temporary exposures to hypoxia, food shortage, and desiccation stress to save on energy expenditure [50]–[52]. In the case of *G. duebeni*, there could be several additional advantages to the observed reductions in summer acclimatised protein synthesis rates in this high intertidal species. First, low protein turnover and maintenance costs have been associated in *Mytilus edulis* with a decrease in sensitivity to temperature change in protein metabolism and rates of oxygen uptake [53], and a greater resistance to weight loss during food limitation [54]. A reduction in maintenance expenditure may also divert energy towards ventilatory and circulatory capacities to ensure a wide thermal tolerance window, and therefore improve survival during thermal extremes [55]. Finally, low protein synthesis rates may also ensure some independence of this rate process from highly fluctuating temperatures to conserve metabolic costs and prolong survival as demonstrated in the semi-terrestrial isopod, *Ligia oceanica*, which also experiences highly fluctuating temperatures in the supra-littoral zone of temperate coastal regions [56]. Despite the presence of extremely low protein synthesis rates in summer acclimatised *G. duebeni*, this study has also revealed that protein synthesis rates are relatively flexible and can increase considerably when the amphipods are acclimated at a common temperature for 4 weeks. These initial observations demonstrate that *G. duebeni* is capable of increasing protein synthesis rates in the laboratory, to the levels more in keeping with those shown by *G. oceanicus* at the same acclimation temperature (Table 3). Moreover, acclimated protein synthesis rates in *G. duebeni* were the same regardless of whether the amphipods were collected from Scotland or Tromsø indicating a lack of any local adaptation or compensation for the changes in temperature with latitude. Clearly the relationship between whole-animal protein synthesis rates and temperature in *G. duebeni* is complex and requires further investigation, especially under natural conditions of temperature variability.

**Table 3:** Whole-animal fractional rates of protein synthesis (*k_s_*) in crustacean species from a range of thermal habitats.

Species	Temperature(°C)	*k_s_* (% day^−1^)	RNA:protein(µg mg^−1^)	*K*_RNA_(mg mg^−1^ day^−1^)	Reference
Acclimated					
*Glyptonotus antarcticus*	0	0.53±0.08	35.75±6.69	0.46±0.13	a
*G. antarcticus*	0	0.32±0.02	22.48±2.96	0.30±0.06	c
*G. antarcticus*	4	0.44±0.06	21.67±2.08	0.42±0.10	c
*Idotea rescata*	4	0.45±0.08	23.98±2.67	0.16±0.03	a
*Saduria entomon*	4	0.57±0.09	10.88±1.00	0.91±0.18	b
*Gammarus duebeni* (70°N)	10	1.88±0.77	6.05±1.22	3.47±1.57	This study
*G. duebeni* (58°N)	10	1.09±0.45	7.36±2.26	1.07±0.45	This study
*G. oceanicus* (58°N)	10	1.72±0.62	5.56±0.51	2.11±0.80	This study
*S. entomon*	13	1.36±0.21	14.36±0.98	1.48±0.70	b
*I.rescata*	14	0.92±0.37	24.37±4.34	0.87±0.37	a
*Macrobrachium bernachii*	20	2.71±0.86	6.01±0.91	7.07±3.38	d
*M. bernachii*	26	2.75±0.68	4.42±0.82	9.82±4.28	d
*Litopanaeus vannamei*	27	4.82	11.12	-	e
*M. bernachii*	30	5.54±1.25	4.28±1.02	14.36±2.42	d
Summer acclimatised					
*Ligia oceanica*	5	0.50±0.21	5.76±0.47	0.76±.29	f
*L. oceanica*	10	0.30±0.05	6.84±0.53	0.52±.10	f
*L. oceanica*	15	0.40±0.04	4.35±0.53	1.11±.14	f
*L. oceanica*	20	0.20±0.04	7.61±0.32	0.28±.06	f
*L. oceanica*	25	0.95±0.59	11±1.09	0.77±.46	f

Fractional rates of protein synthesis (*k_s_*) with associated changes in RNA:protein ratios and RNA activities (*K*_RNA_).

All values scaled to a standard body mass of 1 g wet weight.

Original values taken from: a [8]; b [10]; c [11]; d [58]; e [59]; and f [56].

In the subarctic/boreal species, *G. oceanicus*, protein synthesis rates remained unchanged between populations from Scotland at 13°C and Tromsø at 10°C but fell in the population from Svalbard at 5°C. The decline of protein synthesis rates at the highest latitude suggests that summer acclimatised *G. oceanicus* do not compensate protein synthesis rates at polar latitudes, similar to the lack of any compensation for metabolic rate observed in the same species by Rastrick and Whiteley [39]. Surprisingly the other gammarid species, *G. setosus*, occupying the same shore on Svalbard under the same conditions had protein synthesis levels that were 4.5 higher then those observed in *G. oceanicus.* The reason for this marked difference is unclear but may be related to their respective thermal histories and thermal tolerances, or may even be related to their ability to tolerate other environmental changes. *G. setosus* as an arctic circumpolar, cold-water species is better adapted for living at cold temperatures than *G. oceanicus* which has a more southerly distribution into eurythermal habitats in boreal and cold-temperate regions and can tolerate a wider range of temperatures [27], [36]. *G. oceanicus* also has a different thermal history having survived in southern refugia during the last glacial maxima [22]. Although high rates of protein synthesis have been reported in the embryos and larvae of the Antarctic sea urchin, *Sterechinus neumayeri,* at −1.5°C [57], the consensus in adult marine invertebrates living in the permanently cold waters of the Southern Ocean is that rates of protein synthesis and metabolism are low and associated with a reduction in maintenance costs [4]. Consequently, it is unlikely that the higher rates of protein synthesis observed in *G. setosus* relative to sub-arctic populations of *G. oceanicus* is a cold-water adaptation. Alternatively, it is possible that higher rates of protein synthesis increase the abundance of active ion transporting proteins in the gills of *G. duebeni* enabling their survival in the cooler outlets of freshwater streams [31], [36]. Interestingly, higher rates of protein synthesis in *G. setosus* compared with *G. oceanicus* at the polar latitude are not reflected as differences in life-history traits, as adults from both species produce one well-timed brood per year, and the females take one year to reach the same body size at maturity [26].

Changes in whole-animal fractional rates of protein synthesis in the four gammarid species can be attributed to alterations in either RNA concentrations (RNA:protein) or translational efficiencies (RNA activities, *K*_RNA_). In summer acclimatised *G. locusta,* RNA:protein ratios and RNA activities remained unchanged with latitude despite the 8°C fall in capture temperature. RNA activities, however, were relatively high with *K*_RNA_ in *G. locusta* from Wales at 13°C being 4 times higher than in other crustaceans acclimated at 13/14°C (Table 3). On the other hand, RNA:protein ratios in *G. locusta* of 10–18 µg mg^−1^ were similar to those measured in *G. oceanicus* and in *G. duebeni* at 53 and 58°N, and comparable to those reported previously for other crustacean species acclimated at similar temperatures (Table 3). Natural populations of the warm-temperate gammarid species appear to maintain relatively high rates of whole-animal protein synthesis rates across latitudes by conserving *K*_RNA_, which is the first time that this response has been observed at the whole-animal level. Adjustments in *K*_RNA_ have only ever been reported in cell-free systems where protein synthesis rates are allowed to proceed without the restrictions imposed by the supply of nutrients or amino acids [12], [13]. Such adjustments in *K*_RNA_ were thought to occur in species that are naturally adapted to low and constant annual temperatures such as the Antarctic scallop, *Adamussium colbecki* [13], and the Antarctic eelpout, *Pachycara brachycephalum* [12]. However, it appears that alterations in *K*_RNA_ can also occur in temperate species in response to changes in local thermal habitat. The benefit of increasing *K*_RNA_ rather than RNA:protein in *G. locusta* as latitudinal temperatures fall is unknown but may be related to the energetic costs associated with a temperature–related increase in RNA turnover rates.

In *G. duebeni*, the relatively low summer acclimatised rates of protein synthesis across all three latitudes were matched by low RNA activities but a large increase in RNA content between Wales and Tromsø, which is the primary mechanism for cold-compensation of protein synthesis rates in eurythermal ectotherms [4]. The maintenance of low protein synthesis rates in summer acclimatised *G. duebeni* from Tromsø despite a huge increase in RNA:protein further supports the suggestion that the relatively low summer acclimatised protein synthesis rates are transitory and not limited by RNA concentrations. Interestingly, changes in protein synthesis rate in *G. duebeni* after temperature acclimation were brought about by an elevation in *K*_RNA_ at RNA:protein levels more in keeping with the values obtained in tropical prawns (Table 3). In *G. oceanicus*, some compensation for the reduction in temperature at polar latitudes was observed as RNA content increased between populations in Tromsø and Svalbard. However, RNA content was even higher in *G. setosus* from Svalbard showing that cold-compensation of protein synthesis rates is possible in gammarids in the extreme cold. Fraser *et al*. [14] maintain that high concentrations of RNA at higher latitudes are energetically expensive, but it has also been suggested that elevated RNA concentrations are a result of low rates of RNA turnover and enhanced RNA stability, leading to a reduction in maintenance costs [13], [17]. The latter explanation is more likely in *G. setosus* as metabolic rates measured in the same population under the same conditions were low and equivalent to those in *G. oceanicus* [39]. The elevation of RNA content in *G. setosus* to double the values observed in *G. oceanicus* at the same latitude may be a specialised case to ensure survival of *G. setosus* during sea water dilution as mentioned above.

### Conclusion

Summer acclimatised rates of protein synthesis in gammarid amphipods varied between four dominant gammarid amphipod species with different distribution patterns in the northeastern Atlantic and Arctic Oceans. The highest whole-animal protein synthesis rates were observed in the warm-temperate species, *G. locusta*, and the most southerly population of the subarctic/boreal species, *G. oceanicus*. Both species occupy the low intertidal and prefer marine localities and are less tolerant to changes in temperature and salinity than the intertidal species, *G. duebeni*. Rates of protein synthesis were remarkably low in the high intertidal, highly tolerant species, *G. duebeni*, which may be a temporary strategy for saving energy expenditure during extreme summer conditions. Acclimation experiments revealed that *G. duebeni* is capable of increasing protein synthesis rates when held at a constant temperature due to an increase in translational efficiency. In the subarctic/boreal species, *G. oceanicus*, populations from Scotland and Tromsø had similar summer acclimatised protein synthesis rates, but rates declined considerably at the polar latitude. In the cold-water circumpolar species, *G. setosus*, protein synthesis rates were 4.5 fold higher than the rates in *G. oceanicus* at the same polar latitude due to a rise in RNA content. Elevated protein synthesis rates may enable *G. setosus* to hyper-osmoregulate and therefore survive in areas under the influence of cool melt water streams. In general, whole-animal rates of protein synthesis were maintained among populations across latitudes, apart from the polar population of *G. oceanicus* where protein synthesis rates decreased. Although the outcome of the current study is limited by the restricted number of populations used in the study, the current investigation has demonstrated that protein synthesis rates in summer acclimatised gammarid amphipods are complex and influenced by natural thermal gradients and also by the variability of their respective thermal environments. Further investigations are required, especially on the specific effects of variable and extreme temperatures, before we can more fully understand the role of protein synthesis in predicting the ability of gammarid amphipods to survive climate change.
